# Effects of Hydrolysed Whey Proteins on the Techno-Functional Characteristics of Whey Protein-Based Films

**DOI:** 10.3390/ma6030927

**Published:** 2013-03-07

**Authors:** Markus Schmid, Lesley-Virgina Hinz, Florian Wild, Klaus Noller

**Affiliations:** 1 Fraunhofer-Institute for Process Engineering and Packaging IVV, Giggenhauser Strasse 35, Freising 85354, Germany; E-Mails: lesley-virgina.hinz@gmx.de (L.-V.H.); florian.wild@ivv.fraunhofer.de (F.W.); klaus.noller@ivv.fraunhofer.de (K.N.); 2 Chair of Food Packaging Technology, Technische Universität München, Weihenstephaner Steig 22, Freising 85354, Germany

**Keywords:** whey protein isolate, hydrolysed whey protein isolate, barrier properties, mechanical properties, surface tension, yellowish coloration

## Abstract

Pure whey protein isolate (WPI)-based cast films are very brittle due to its strong formation of protein cross-linking of disulphide bonding, hydrogen bonding as well as hydrophobic and electrostatic interactions. However, this strong cross-linking is the reason for its final barrier performance. To overcome film brittleness of whey protein layers, plasticisers like glycerol are used. It reduces intermolecular interactions, increases the mobility of polymer chains and thus film flexibility can be achieved. The objective of this study was to investigate the influence of hydrolysed whey protein isolate (WPI) in whey protein isolate-based cast films on their techno-functional properties. Due to the fact, that the addition of glycerol is necessary but at the same time increases the free volume in the film leading to higher oxygen and water vapour permeability, the glycerol concentration was kept constant. Cast films with different ratios of hydrolysed and not hydrolysed WPI were produced. They were characterised in order to determine the influence of the lower molecular weight caused by the addition of hydrolysed WPI on the techno-functional properties. This study showed that increasing hydrolysed WPI concentrations significantly change the mechanical properties while maintaining the oxygen and water vapour permeability. The tensile and elastic film properties decreased significantly by reducing the average molecular weight whereas the yellowish coloration and the surface tension considerably increased. This study provided new data which put researchers and material developers in a position to tailor the characteristics of whey protein based films according to their intended application and further processing.

## 1. Introduction

The primary function of a food packaging is the protection of the food product against external influences especially against oxygen and/or water vapour [1,2,3,4]. This function is often provided by petroleum-based polymers like EVOH (ethylene-vinyl-alcohol) which acts as an oxygen barrier material in multilayer structures for achieving high barrier properties. According to the GVM (Gesellschaft für Verpackungsmarktforschung) the use of EVOH as barrier material in multilayer materials will be approximate 645 km^2^ in the year 2014 in Germany. Hence, EVOH will be the most important barrier material in multilayers although EVOH is neither renewable nor biodegradable [4,5]. To focus more on sustainability and recyclability there is an increase of research activities on renewable materials providing similar barrier and mechanical properties in order to replace petroleum-based materials and to increase the recyclability of multilayer films. A possible alternative to achieve suitable mechanical as well as high oxygen and water vapour barrier properties is coating of polymer films with whey protein, which is a by-product of cheese manufacturing [6,7,8,9]. As whey protein layers are able to act as an excellent oxygen barrier material and provide suitable mechanical properties, they could be a substitution for an EVOH layer. Whey proteins are soluble in water and can be removed chemically or enzymatically from the polymer substrate resulting in an increasing mono-fraction recyclability of multilayer [10]. According to the International Whey Conference of 2008 there are 50 million tons of whey produced in Europe where of 40% are discarded. The use of whey proteins as barrier material in multilayer structures could increase the attractive value of whey and new branches of production in the cheese manufacturing and polymer processing industry could be established and in addition could contribute to sustainability [11].

Unplasticised whey protein based films are brittle and therefore not applicable for industrial application. Therefore the addition of glycerol as plasticiser is performed in order to overcome film brittleness and to achieve film flexibility. Plasticizers are low-molecular-weight substances, which decrease the protein-protein interaction and increase the mobility of polymer chains additionally lead to a higher free volume in the protein network [12,13,14]. Hence, plasticizers are able to increase the oxygen and water vapour permeability which is not favourable for most sensitive food products which have to be protected against oxygen and/or water vapour [12,15,16,17,18].

To investigate the influence of an alternative low-molecular-weight substance on the mechanical properties, hydrolysed WPI were incorporated in high molecular weight WPI formulations and characterised accordingly. Hydrolysed WPI contains shorter chains of amino acids providing a higher number of polar end groups than unhydrolysed WPI and has a lower molecular weight (M_W_) [14].

The major objective of this study was to determine the effect of hydrolysed WPI in whey protein isolate based cast films on their respective techno-functional properties, namely water vapour and oxygen barrier, tensile and elastic film properties, yellowish coloration and the surface tension. The novelty of this approach is given by the fact that mixtures of WPI and fully hydrolysed WPI were analysed in contrast to former studies where either fully or partially hydrolysed films were analysed [13,14]. Thus, this study is of relevance since films from mixtures of WPI and hydrolysed WPI may have different properties than films entirely produced from hydrolysed WPI. This approach is also interesting from an economical point of view, since the addition of fully hydrolysed WPI in suitable amounts is more economical than hydrolysing the whole WPI composition in WPI based barrier films for packaging applications.

Due to the fact that the addition of glycerol is necessary but at the same time increases the free volume leading to higher oxygen and water vapour permeability [15,16,19,20], the glycerol concentration was kept constant. Cast films with different ratios of hydrolysed and not hydrolysed WPI were produced. They were characterised in order to determine the influence of the lower molecular weight caused by the addition of hydrolysed WPI on the above-mentioned techno-functional properties. This study provides new data which put researchers and material developers in a position to tailor the characteristics of whey protein based films according to their intended application and further processing.

## 2. Materials and Methods

### 2.1. Materials

The whey protein isolate (WPI) BiPro with a protein content on dry basis (*N* × 6.38) of 95% and the hydrolysed whey protein isolate BiZate 3 with a degree of hydrolysis (DH) of 10% and a protein content on dry basis (*N* × 6.38) of 94% by Davisco Foods International (Le Sueur, Minnesota) were used to produce the whey protein isolate based films. Data for degree of hydrolysis and protein content were provided by Davisco Food International. To overcome film brittleness glycerol (Gly) as plasticizer supplied by Merch Schuchardt OHG (Hohenbrunn, Germany) was used.

### 2.2. Film Casting

10% WPI aqueous solution (pH 7) was heated at 90 °C and stirred for 30 min in an electric stirrer, for protein denaturation, accordingly to Mc Hugh and Krochta [21] and Schmid *et al.* [2]. The flask volume of the electric stirrer was 1.5 L and the volume produced per sample batch was 1 L. After the WPI solution was cooled to room temperature, foam and bubbles were removed in an ultra-sonic bath (frequency of 37 kHz) by using a pipette after 15 min of ultra-sonic treatment. Gly (66.7% (w/w) based on dry matter basis of BiPro) was added and the WPI solution stirred for 30 min in a magnetic stirrer (200 rpm). Finally the WPI solution was treated in an ultra-sonic bath for 15 min to remove again the bubbles.

To analyse the effect of hydrolysed WPI content at constant amounts of Gly on film properties, unhydrolysed WPI was substituted by hydrolysed WPI. Thus 1% (w/w) BiPro was substituted by 1% (w/w) BioZate 3. This means that cast films with different ratios of hydrolysed (BioZate3) and unhydrolysed WPI (BiPro) were produced whereas the Gly concentration was kept constant. This ratio is displayed by the *x*-axis labelling of the figures.

A volume 17.1 mL of WPI formulations was filled into a petri dish (120 mm × 120 mm × 14.5 mm) made of polystyrene to form films with a film thickness of approximate 200 µm. To achieve a homogenous distribution of film thicknesses eight-shaped movements with the petri dish on a water-levelled ground were performed. The WPI films were cured at 23 °C and 50% relative humidity (RH) for approximate 7 days until they reached the equilibrium moisture content at the given conditions. The main advantages of cast films are the independency of any substrate for characterisation and evaluation of the results. In contrast to that it is not possible to directly compare the results to materials applied by commonly used coating techniques since no polymer orientation and a significantly slower curing takes place. However, it is applicable to compare the results within the same series of trials as performed in this study.

### 2.3. Water Vapour Transmission Rate Measurement

The gravimetric screw cup method according to DIN 53 122-1 was used to measure the WVTR (water vapour transmission rate) of the cast films at 23 °C and 50% → 0% RH.

The WVTR is calculated by the following equation:

(1) $$\text{WVTR}=\frac{24}{t}\times \frac{\text{Δ}m}{A}\times 10^{4}$$

Where *t* is the period of time between two weight measurements in h, Δ*m* represents the weight difference between two weight measurements in g and A is the test area in cm^2^. The WVTR values are stated in the unit g/(m^2^ d) and converted to a thickness of 100 µm (Q_100_) in order to allow direct comparison of different materials independently of the coating thickness.

A fourfold determination was performed in all cases [22].

### 2.4. Oxygen Transmission Rate Measurement

The OTR (oxygen transmission rate) measurement was performed according to DIN 53380-3 at 23 °C and 50% RH by an instrument of Brugger Feinmechanik GmbH. The WPI films are masked using aluminium films in order to stabilise the samples. In case of deviation of >10% between two OTR values a third determination is used. The OTR values are given in the unit cm^3^ (STP)/(m^2^ d bar) and were converted to a thickness of 100 µm (Q_100_) in order to allow direct comparison of different materials independently of the film thickness [23].

### 2.5. Tensile Property Measurement

The tensile strength (TS) of WPI-based films was measured by a universal compression-tension testing machine (Model RM 50 by Doli GmbH Industrie Electronic, München, Germany). According to DIN EN ISO 527 forces were applied to the samples from both sides until a change in shape or a fracture takes place [24].

The WPI films were cut into strips of 15 mm width and a length of 70 mm. In order to measure the tensile properties as a function of the thickness, the thickness of WPI films were measured. For each specimen fivefold determinations were performed and the arithmetic average and standard deviation were calculated.

The ends of the specimen were clamped by utilizing pneumatic grips in the loading frame. The initial gauge length of the specimen was set to 50 mm. After the traverse stroke and the force were tarred, the specimen was subjected by an applied force using a load cell of 50 N. The specimen was stretched using a testing speed of 100 mm/min. A tenfold determination was performed for each sample at testing conditions of 23 °C and 50% RH.

### 2.6. Young’s Modulus Measurement

The Young’s Modulus or Elastic Modulus (EM) of WPI films were measured by a universal compression-tension testing machine (Model RM 50 supplied by Doli GmbH Industrie Electronic, München, Germany) in a separate measurement next to the tensile test. According to DIN EN ISO 527 a sample is subjected to applied forces until an elastic deformation takes place [24]. The Young’s Modulus is the proportional ratio of stress to strain in the elastic region [25,26]. Stripes of 15 mm width and 70 mm length were used. The thickness of every specimen is measured at five different positions and the arithmetic average and standard deviation are determined in order to measure the elastic behaviour of WPI films as function of the thickness. The specimens were subjected to applied forces using a load cell of 50 N. An initial gauge length of 50 mm was set. The specimens were stretched using a testing speed of 0.5 mm/min at constant testing condition of 23 °C and 50% RH. Tenfold determination was performed.

### 2.7. Surface Energy Measurement

The surface energy of WPI films was measured by the contact angle measuring system (Model G2, Krüss GmbH, Stephanskirchen/Rosenheim, Germany) using the sessile drop method. This method applies a liquid static drop on the surface of a solid to determine the contact angle between the baseline of the drop and the tangent at the drop boundary.

After the glass syringes were refilled by the following testing liquids: double distilled water, diiodo-methane, ethylene-glycol and dimethlphthalat, the sample are cut into pieces (7.5 mm × 7.5 mm). The samples were positioned on the moveable suction plate which is connected with a vacuum pump and fixed using a tape. A testing drop of 3 µL is induced on the surface of the solid using a dosing rate of 20 µm/min in order to set an ideal screen of the liquid drop at the monitor by help of contrasts, lightning, sharpness, screen size and external mirror image of the liquid drop. After an ideal screen drop was set, the screen of the inducing liquid drop on the solid surface was freezed to measure the contact angle between the baseline which is adapted to the liquid drop position and the tangent at the drop boundary. The contact angle of each testing liquid was measured at five different positions on the solid surface of 5 replicates. The arithmetic average and the standard deviation were calculated. According to Young’s equation the surface energy can be calculated as follows:

(2) $$\sigma_{s}=\gamma_{\text{sl}}+\sigma_{l}\cdot \cos\theta$$

Where σ_s_ is the surface tension of the solid in mN/m, σ_l_ stands for the surface tension of the liquid in mN/m, γ_sl_ is the interfacial tension between the solid and the liquid in mN/m and cosθ is the contact angle between surface tension of liquid and the interfacial tension between liquid and solid in angular degree. According to Owens, Wendt, Rabel und Kaelble method the surface energy is divided into disperse and polar fractions which were calculated accordingly.

### 2.8. Colour Measurement

The colour measurement of WPI films is performed by a spectrophotometer (CM-700d, Konica Minolta Sensing, Tokio, Japan). First of all the spectrophotometer is calibrated by use of a white and black device. Using an illuminant D65 and a wavelength range from 400 to 700 nm a white ceramic plate is measured as reference. The colour was measured at five replicates (films). The SCE (Specular Component Excluded) modus was adjusted.

### 2.9. Film Thickness

The film thicknesses of WPI films were measured by a Precision Thickness Gauge (Model FT3 by Rhopoint Instruments, Bexhill on Sea, UK) at five different positions. The arithmetic average of the film thicknesses are used to determine OTR, WVTR and mechanical film properties, whereas the standard deviations were taken into account for the error propagation accordingly.

### 2.10. Residual Water Content Measurement

To measure the residual water content of WPI films a scale (MA30, Sartorius AG, Göttingen, Germany) was used. The IR-sample dish made of aluminium is tared and 0.5 g of the WPI film is placed into it and located plane. The sample is dried at a temperature of 105 °C until the mass is constant for at least 5 min. The water content is given in percent. For every sample a threefold determination was performed.

### 2.11. Statistical Analysis

A completely randomized experimental design was used. Thus, the run sequence of the experimental units was determined randomly. The randomisation was performed by the computer programme Visual-XSel 12.0 Mulivar (CRGRAPH, Munich, Germany). Statistical analyses were performed with the Kolmogorov-Smirnov-Test procedure in Visual-XSel 12.0 Multivar and level of significance was defined at 5%. This test was applied in order to investigate if a normal distribution adequately describes the sets of data which were achieved. In all cases the hypothesis of normality was validated. Thus, calculated standard deviations which are given as error bars in the figures can be reasonably interpreted. The mean values for OTR were calculated from twofold determinations. Those data were not tested on normal distribution but the minimum and maximum values are given accordingly.

## 3. Results and Discussions

### 3.1. Water Vapour Transmission Rate

**Figure 1:**
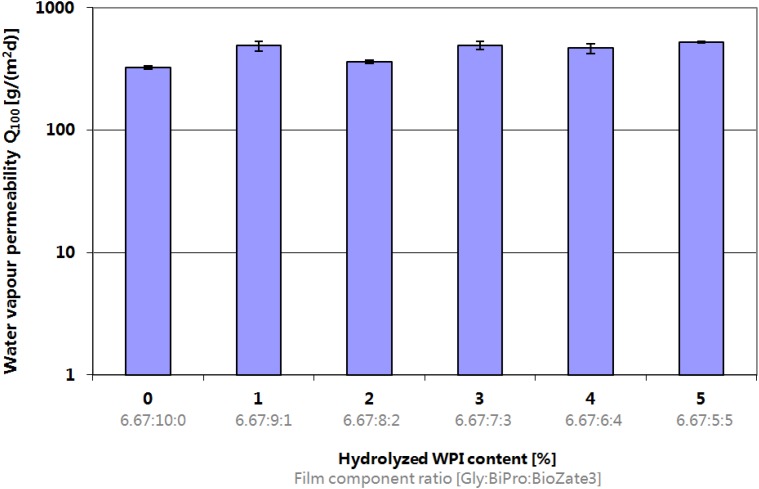
Influence of hydrolysed whey protein isolate (WPI) content and film component ratio in denatured WPI-based films on Water Vapour Permeability.

There was no significant difference in WVTR values of WPI-based films if M_W_ reduction takes place (Figure 1). These results are consistent with Sothornvit and Krochta [14] who analysed the effect of hydrolysed WPI at equal amount of plasticizer on WVTR values. However, the overall values determined in this study are not comparable to the values published by Sothornvit and Krochta [14] since another method at (ASTM E96-92) was used. Perez-Gago *et al.* [27] postulated similar results between native and denatured WPI films and Mate and Kochta [28] on WVTR between WPI and β-lactoglobulin films, which showed that the whey protein structure did not influence the WVTR. Based on the WVTR results of this study which are consistent with and complementary to other publications it can be concluded that the effect of hydrolysed WPI in WPI-based films does not significantly influence or increase the WVTR as added plasticizer. Since increasing permeability is undesirable for sensitive food quality it could be a better approach to reduce the plasticizer concentration to decrease the WVTR than addition of hydrolysed WPI, whereas film flexibility has to be taken into consideration.

### 3.2. Oxygen Transmission Rate

**Figure 2:**
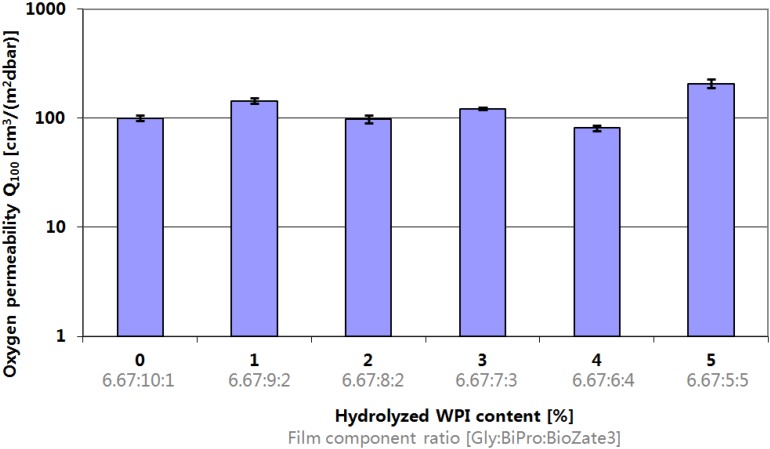
Influence of hydrolysed WPI content and film component ratio in denatured WPI-based films on Oxygen Permeability.

Oxygen can induce the oxidation of fatty food products which influences negatively the food product and makes it unenjoyably for the consumer. In WPI-based films there is no coherence observable between the reducing M_W_ and the OTR values (Figure 2). According to the results of Sothornovit and Krochta [13] who analysed the impact of hydrolysed WPI content at equal amount of plasticizer on OTR leads to the results that there are little or no effects of hydrolysed WPI content on OTR values. Hence, the results are consistant with Sothornvit and Krochta and can be confirmed. Thus, the addition of hydrolysed WPI has little or no effect on film OTR values. In addition to that, the results confirm that the WPI composition and structure has no significant effect on film permeability as confirmed by several previous publications [10,27,28,29]. However, the approach of this work has to be differentiated from those publications since in this study hydrolysed and not hydrolysed WPI were mixed in contrast to the utilisation of hydrolysed WPI with different degree of hydrolysis (DH). Nevertheless, the results of this study confirm that the final effect of the current approach is the same.

### 3.3. Tensile Property

**Figure 3:**
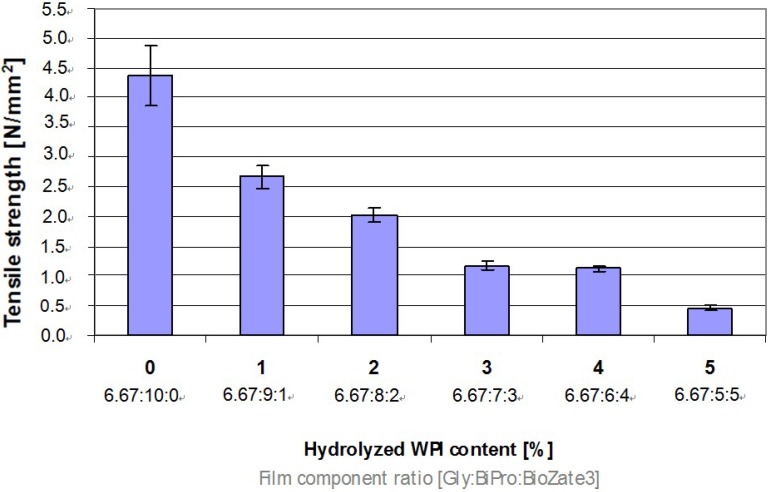
Influence of hydrolysed WPI content and film component ratio in denatured WPI-based films on Tensile strength.

Tensile properties of films are important for handling processes during processing and distribution of the products in order to protect the product against mechanical damages. In addition, the tensile properties provide data for the quantification of protein network interactions. In WPI-based films TS decreases significantly when the M_W_ decreases (Figure 3). These results can be explained by weaker protein network interactions between the shorter chains of hydrolysed WPI in comparison to the longer chains of unhydrolysed WPI. Thus, hydrolysed WPI might also able to act as internal plasticizer. Therefore with increasing hydrolysed WPI content and equal amount of Gly the protein-protein interactions decreases and the mobility of polymer chains increases leading to high plasticizing effects in WPI-based films.

### 3.4. Elastic Property

**Figure 4:**
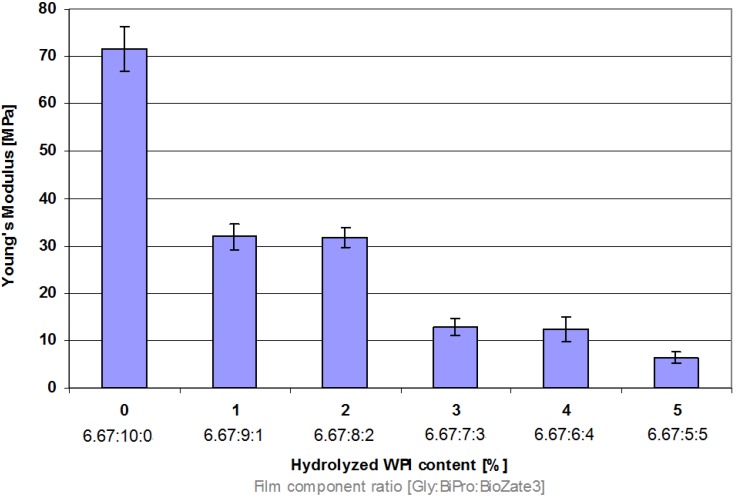
Influence of hydrolysed WPI content and film component ratio in denatured WPI-based films on Young’s Modulus.

Elastic properties of films are also important for example during stacking of products. A material becomes stiffer or in other words less flexible the higher the Young’s Modulus (EM) value of the material is [30]. In WPI-based films the Young’s Modulus (EM) significantly decreases if the M_W_ is reduced (Figure 4) leading to less stiff and more flexible films. According to literature with increasing shorter chains of amino acids by increasing the hydrolysed WPI content the flexibility increases [10,13]. In this case it can be explained by the increasing hydrolysed WPI content because hydrolysed WPI forms weaker intermolecular forces and thus EM decreases by higher hydrolysed WPI ratios leading to more flexible films.

### 3.5. Surface Energy

**Figure 5:**
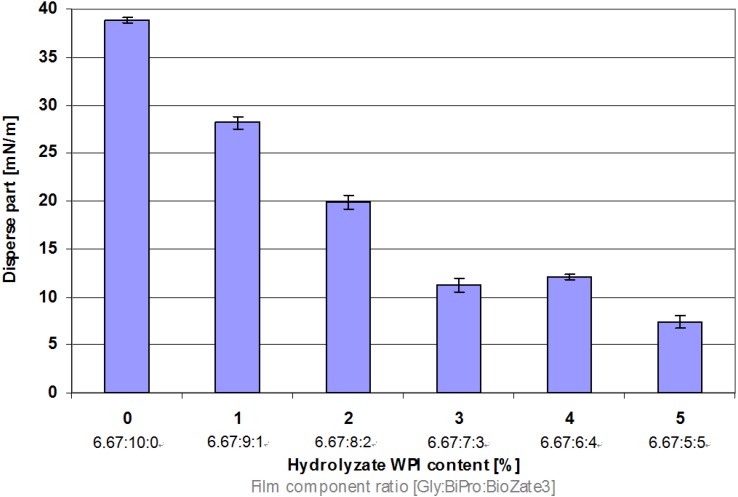
Influence of hydrolysed WPI content and film component ratio in denatured WPI-based films on disperse part of surface energy.

For industrial coatings the knowledge of surface energies and in particular the polar and disperse part of the surface energy of solids which are going to be coated or printed is an important factor. In the WPI-based films of this study the disperse part of the surface energy decreases significantly if the M_W_ is reduced (Figure 5). This behaviour of WPI-based films can be explained by the van-der-Waals forces which occur in dispersive materials [31]. The lower the M_W_, the lower are the van-der-Waals forces along the polymer chains. This is due to the fact that the molecular weight is roughly proportional to the number of electrons in the molecule, usually about twice the number of electrons. Since the van-der-Waals forces between molecules increase with increasing the number of electrons per molecule the van-der-Waals forces increase with increasing molecular weight and *vice versa* [32].

**Figure 6:**
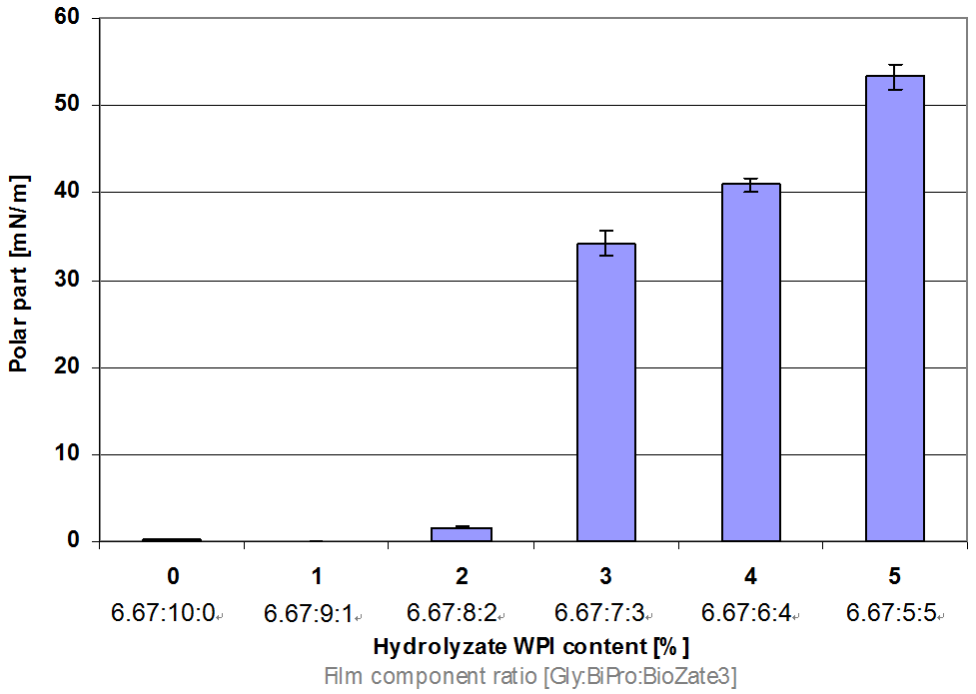
Influence of hydrolysed WPI content and film component ratio in denatured WPI-based films on polar part of surface energy.

As the disperse part decreases the polar part of the surface energy increases significantly if the M_W_ decreases (Figure 6). This can be explained by the increasing amount of polar end groups. Hydrolysed WPI contain shorter chains of amino acid chains and thus provide a higher amount of polar end groups than unhydrolysed WPI. The higher the hydrolysed WPI content in denatured WPI-based films, the higher is the amount of polar end groups.

### 3.6. Yellow Colouration

Packaging must provide attractiveness to the consumer, therefore optical properties of WPI-based films are analysed. Most favourable are highly transparent materials without any coloration [2].

**Figure 7:**
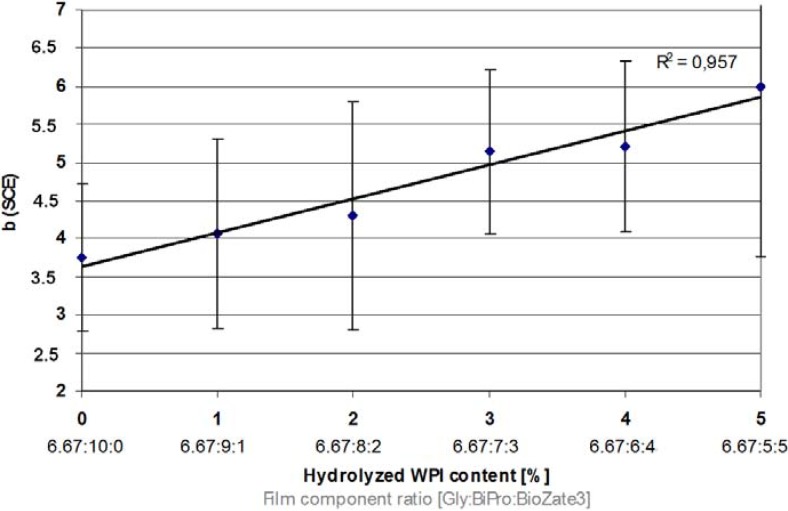
Influence of hydrolysed WPI content and film component ratio in denatured WPI-based films on yellow colouration.

The results show that when M_W_ decreases the yellow colouration of WPI-based films increase slightly (Figure 7). This marginally increasing yellow colouration might be related to higher amounts of yellowish whey metabolites in BioZate 3 which are either not present in BiPro or are present in smaller amounts.

## 4. Conclusions

This study shows that increasing hydrolysed WPI concentrations significantly change the mechanical properties while maintaining the oxygen and water vapour permeability. The tensile and elastic film properties decrease significantly by reducing the average molecular weight whereas the yellowish coloration and the surface tension considerably increase. This study provides new data which put researchers and material developers in a position to tailor the characteristics of whey protein isolate based films according to their intended application and further processing.

## References

[1] Adegboye T., Somade S. (2009). 111 Question and Answers in Packaging Technology.

[2] Schmid M., Dallmann K., Bugnicourt E., Cordoni D., Wild F., Lazzeri A., Noller K. (2012). Properties of whey protein coated films and laminates as novel recyclable food packaging materials with excellent barrier properties. Int. J. Polym. Sci..

[3] Sängerlaub S., Gibis D., Kirchhoff E., Tittjung M., Schmid M., Müller K. (2013). Compensation of pinhole defects in food packages by application of iron-based oxygen scavenging multilayer films. Packag. Technol. Sci..

[4] Schmid M., Benz A., Stinga C., Samain D., Zeyer K.P. (2012). Fundamental investigations regarding barrier properties of grafted PVOH layers. Int. J. Polym. Sci..

[5] Buchert J., Ercili Cura D., Ma H., Gasparetti C., Monogioudi E., Faccio G., Mattinen M., Boer H., Partanen R., Selinheimo E., Lantto R., Kruus K. (2010). Crosslinking food proteins for improved functionality. Ann. Rev. Food Sci. Technol..

[6] de Wit J.N. (2001). Lehrbuch der Molke und Molkenerzeugnisse.

[7] Schmid M., Held J., Wild F., Noller K. (2011). Thermoforming of whey protein-based barrier layers for application in food packaging. Food Sci. Technol..

[8] Schmid M. (2012). Whey protein-based coatings as sustainable barrier material in food packaging applications. Proceeding of IAPRI World Packaging Conference 2012.

[9] Hernandez-Izquierdo V.M., Krochta J.M. (2009). Thermal transitions and heat-sealing of glycerol-plasticized whey protein films. Packag. Technol. Sci..

[10] Whitaker J.R., Shahidi F., Munguia A.L., Yada R.Y., Fuller G. (1998). Functional Properties of Protein and Lipids.

[11] Bugnicourt E., Schmid M., Nerney O.M., Wildner J., Smykala L., Lazzeri A., Cinelli P. (2013). Processing and validation of whey protein coated films and laminates at semi-industrial scale as novel recyclable food packaging materials with excellent barrier properties. Adv. Mater. Sci. Eng..

[12] Hong S.I., Krochta J.M. (2003). Oxygen barrier properties of whey protein isolate coatings on polypropylene films. J. Food Sci..

[13] Sothornvit R., Krochta J.M. (2000). Oxygen permeability and mechanical properties of films from hydrolyzed whey protein. J. Agric. Food Chem..

[14] Sothornvit R., Krochta J.M. (2000). Water vapor permeability and solubility of films from hydrolyzed whey protein. J. Food Sci..

[15] McHugh T.H., Krochta J.M. (1994). Sorbitol-plasticized *vs*. glycerol-plasticized whey-protein edible films—Integrated oxygen permeability and tensile property evaluation. J. Agric. Food Chem..

[16] McHugh T.H., Aujard J.F., Krochta J.M. (1994). Plasticized whey-protein edible films—Water-vapor permeability properties. J. Food Sci..

[17] Hong S.I., Krochta J.M. (2004). Whey protein isolate coating on LDPE film as a novel oxygen barrier in the composite structure. Packag. Technol. Sci..

[18] Perez-Gago M.B., Krochta J.M., Gennadios A. (2002). Formation and Properties of Whey Protein Films and Coating. Protein-based Films and Coatings.

[19] Onwulata C., Huth P. (2008). Whey processing, functionality and health benefits. Variation: IFT Press Series.

[20] Yoo S., Lau S.H., Krochta J.M. (2012). Grease penetration and browning resistance of pulpboard and paperboard coated with whey protein. Packag. Technol. Sci..

[21] McHugh T.H., Krochta J.M. (1994). Water-vapor permeability properties of edible whey protein-lipid emulsion films. J. Am. Oil Chem. Soc..

[22] DIN 53122-1 (2001). Bestimmung der Wasserdampfdurchlässigkeit. Prüfung von Kunststoff-Folien, Elastomerfolien, Papier, Pappe und anderen Flächengebilden.

[23] DIN 53380-3 (1998). Bestimmung der Gasdurchlässigkeit. Sauerstoffspezifisches Trägergas-Verfahren zur Messung an Kunststoff-Folien und Kunststoff-Formteilen.

[24] DIN EN ISO 527-1 (1996). Bestimmung der Zugeigenschaften. Allgemeine Grundsätze.

[25] Davis J.R. (2004). Tensile Testing.

[26] Beer F., Russell J.E. (1981). Mechanics of Materials.

[27] Perez-Gago M.B., Nadaud P., Krochta J.M. (1999). Water vapor permeability, solubility, and tensile properties of heat-denatured *versus* native whey protein films. J. Food Sci..

[28] Mate J.I., Krochta J.M. (1996). Comparison of oxygen and water vapor permeabilities of whey protein isolate and beta-lactoglobulin edible films. J. Agric. Food Chem..

[29] Perez-Gago M.B., Krochta J.M. (1999). Water vapor permeability of whey protein emulsion films as affected by pH. J. Food Sci..

[30] Endres H.-J., Siebert-Raths A. (2011). Engineering Biopolymers: Markets, Manufacturing, Properties and Applications.

[31] Drummond C.J., Chan D.Y.C. (1997). van der Waals interaction, surface free energies, and contact angles: Dispersive polymers and liquids. Langmuir.

[32] Pauling L. (1988). General Chemistry.

